# Symbiont evolutionary history underpins quality to an insect host

**DOI:** 10.1093/jisesa/ieag080

**Published:** 2026-07-29

**Authors:** Audrey K Miller, Ava V Virtuoso, Kayla S Stoy

**Affiliations:** Department of Biological Science, Florida State University, Tallahassee, FL, 32304, USA; Department of Biological Science, Florida State University, Tallahassee, FL, 32304, USA; Department of Biological Science, Florida State University, Tallahassee, FL, 32304, USA

**Keywords:** symbiosis, mutualism, Coreidae

## Abstract

While many insects have evolved mechanisms to ensure the faithful transmission of bacterial symbionts across generations, the majority of insects lack such mechanisms. Instead, many insects must acquire symbionts from their local environments. Without mechanisms for vertical transmission, insects are thought to be at risk of experiencing low fitness by acquiring low-quality symbiont strains. For example, insects dependent on environmental symbiont transmission may acquire nonsymbiotic bacteria or symbionts that are adapted to other co-localized hosts. Yet, insects often appear capable of interacting with a wide variety of symbiont strains, and environmental transmission is prevalent across diverse insect species. Therefore, it remains unclear whether the evolutionary history of a symbiont actually impacts its benefit to hosts. Here, we test whether the recent evolutionary history of a symbiont impacts its quality to an insect host by inoculating *Anasa tristis* De Geer (Hemiptera: Coreidae) insects with Burkholderiaceae strains isolated from a conspecific host, a distantly related heterospecific host, and from soil. We find that the soil strain offers few fitness benefits. In contrast, both strains with a recent history of host association support insect survival to adulthood, but the greatest benefits emerge from the symbiont isolated from a conspecific host, which also improves insect development rates. Overall, our study demonstrates that the evolutionary history of a symbiont may impact its quality to insect hosts and lays a foundation for future work to further examine whether environmental transmission carries costs for insect hosts.

## Introduction

Insects are notorious for their dependence on microbial symbionts for essential fitness functions. Microbial symbionts provide insects access to nutrition, defense against pathogens, and increased resilience to abiotic stress (Braendle et al. 2003, Currie et al. 2003, Kaltz and Koella 2003, Oliver et al. 2003, Hosokawa et al. 2007, Kaltenpoth et al. 2010, Brumin et al. 2011, Gerardo et al. 2020, Kaltenpoth and Flórez 2020, Berasategui et al. 2022, Cornwallis et al. 2023, García-Lozano et al. 2024). Association with microbial symbionts underpins insect niche expansion and has promoted the adaptive radiation of insect species (Cornwallis et al. 2023, García-Lozano et al. 2024). Insects and their microbial symbionts have repeatedly become irreversibly dependent on one another, such that neither can survive without the other. Such obligate symbiosis has independently evolved at least 16 times across 89 insect families (Cornwallis et al. 2023), suggesting the phenomenon of symbiotic dependence is widespread across diverse insect species.

Across obligate insect symbioses, a plethora of mechanisms have evolved to ensure the faithful transmission of microbial symbionts across generations (Bright and Bulgheresi 2010, Salem et al. 2015). Through these transmission mechanisms, parents ensure that their offspring acquire necessary symbionts, eliminating the risk that symbionts will go unacquired or that offspring acquire low-quality partners that lack symbiotic benefit. For example, transovarial symbiont transmission to offspring is common among Hemiptera, including the well-known example occurring in aphids (Braendle et al. 2003, Bennett and Moran 2015).

Remarkably, however, most insect symbioses do not conform to this model. Rather, many insects that require symbionts for essential fitness functions acquire these symbionts from their local environments (Salem et al. 2015, Chomicki et al. 2020, Kaltenpoth and Flórez 2020). Within these associations, there are few mechanisms for ensuring the consistent transmission of beneficial symbionts across generations. This is thought to be costly for hosts that risk experiencing reduced fitness by acquiring low-quality symbionts from their environments (Frank 1996, Herre et al. 1999, Bronstein 2001, Chomicki et al. 2020). However, environmentally acquired insect symbioses are common. Is environmental symbiont transmission costly?

For evolutionary costs to arise under environmental transmission, insects must encounter symbionts that vary in quality. When symbionts are acquired from the environment rather than vertically inherited, insects may associate with a diverse pool of closely related microbes that differ in their evolutionary histories. For example, hosts may acquire microbial strains that are closely related to established symbionts but are themselves nonsymbiotic, or they may acquire strains that have evolved in association with a heterospecific insect host. Here, we test whether a symbiont’s recent evolutionary history influences its quality to an insect host. Specifically, we leverage Burkholderiaceae symbionts isolated from insect hosts and soil to examine whether symbionts with a recent history of selection in these distinct environments vary in their effects on the fitness of the squash bug *Anasa tristis* De Geer (Hemiptera: Coreidae).

Heteropteran insects, including *A. tristis*, depend on bacterial symbionts in the Burkholderiaceae family for development and survival (Kaltenpoth and Flórez 2020, Acevedo et al. 2021). Insects primarily benefit from *Caballeronia* strains, but *Paraburkholderia* strains can also provide fitness benefits (Gordon et al. 2016, Acevedo et al. 2021, Sullivan et al. 2025). Symbionts are harbored in the M4 region of the insect hindgut, which serves as a symbiotic organ (Kaltenpoth and Flórez 2020, Kikuchi et al. 2020, Junker et al. 2025). These microbial partners provision essential nutrients deficient in the phloem-feeding diet of their heteropteran hosts (Martinez et al. 2024, Mendiola et al. 2024).

*Anasa tristis* insects geographically overlap with a wide variety of closely related heteropteran species that all depend on Burkholderiaceae symbionts (Packauskas 2010, Stoy et al. 2023). *Anasa tristis* insects often harbor genetically diverse symbiont communities with substantial taxonomic overlap with co-localized congeners (Stoy et al. 2023). *Anasa tristis* can acquire symbionts from conspecific fecal matter (Villa et al. 2023) and from soil. Closely related co-localized heteropteran hosts also acquire symbionts from soil, and the presence of heteropterans in a local environment can enrich symbionts in the soil (Parajuli et al. 2026). Together, these dynamics suggest that *A. tristis* insects are likely exposed to symbionts with diverse evolutionary histories.

We hypothesize that insects receive the greatest fitness benefits from symbionts that have a recent evolutionary history of association with closely related insect hosts. We test this hypothesis by inoculating *A. tristis* insects with Burkholderiaceae strains isolated from a conspecific host, a heterospecific host, and soil. We find that symbionts with distinct evolutionary histories vary in their quality to hosts, with the greatest benefits emerging from symbionts that have a recent history of host association.

## Methods

### Symbiont Strains

A conspecific strain (GAOX1) of *Caballeronia* sp. (Burkhol­deriales: Burkholderiaceae) was previously isolated from an adult *A. tristis* squash bug in Georgia, United States (Acevedo et al. 2021, Stoy et al. 2023). We isolated a heterospecific *Paraburkholderia* sp. strain (LS1) (Burkholderiales: Burkholderiaceae) from the M4 symbiotic organ of an adult *Largus succinctus* (Hemiptera: Largidae) collected in Tallahassee, Florida. A soil strain (Snap2a) of *Caballeronia* sp. was previously isolated from bulk soil at a field site where heteropterans are common in Georgia, United States (Garcia et al. 2014). This strain was selected due to its recent evolutionary history in soil. We note that because these symbionts are transmitted through the environment, each of these strains may have experienced selection across each of the selective environments assessed in this study (eg soil, conspecific hosts, or heterospecific hosts) at some point in their evolutionary histories. As such, in this study, we examine whether symbiont strains with the most recent evolutionary history in each of these environments vary in their effects on insect fitness. Strain identity was assessed using 16S rRNA gene sequencing (PCR with universal primers 27F and 1492R followed by Sanger sequencing) and the NCBI BLAST tool. We further confirmed strain identity using a phylogenetic approach (Supplementary Fig S1; Supplementary Table S1).

*Largus succinctus* geographically overlaps with *A. tristis* but differs in diet. While *A. tristis* specializes on cucurbit plants, *L. succinctus* feeds primarily on pine and other woody plants. Because members of the Burkholderiaceae function as nutritional symbionts, differences in host diet are expected to impose distinct selective pressures on symbiont function. Prior work also suggests that Largidae insects harbor Burkholderiaceae strains that are genetically distinct from those harbored by Coreidae (Gordon et al. 2016). We therefore included a symbiont isolated from *L. succinctus* to test whether symbionts associated with divergent hosts differ in their effects on host fitness.

### Fitness Assays

To examine the effect of symbiont evolutionary history on insect fitness, we inoculated second instar *A. tristis* nymphs with conspecific strain GAOX1 (*n* = 49), heterospecific strain LS1 (*n* = 53), and soil strain Snap2a (*n* = 62). Insects inoculated with water served as aposymbiotic controls (*n* = 57). Inoculations were conducted using aqueous feeding solutions (∼10^7^ cells/ml), as described in Stoy et al. (2023). Nymphs were exposed to feeding solutions for 24 h before they were replaced with surface-sterilized zucchini. Survival and development rates were monitored every other day, and zucchini was replaced at this time. Inoculations and data collection were independently replicated two times per treatment. Number of bacterial colony forming units (CFUs) per adult M4 was determined by dissecting insects, homogenizing the M4, serial diluting, and plating on yeast glucose. Development rate to adulthood and survival probability were analyzed using mixed effects cox proportional hazard models with symbiont as a fixed effect and replicate as a random effect (Therneau 2024). Pairwise comparisons between treatments were conducted using estimated marginal means with the R package emmeans (Lenth 2022). A chi-squared test was used to analyze survival proportions. Statistical analyses were conducted in R version 4.2.2.

## Results

**Fig. 1.**
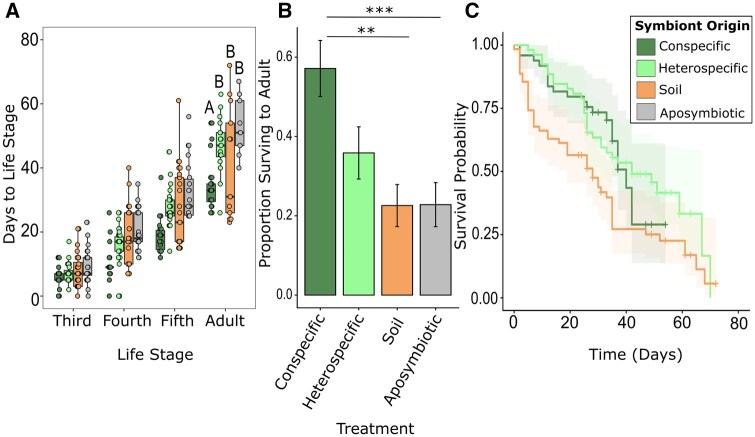
Effect of symbiont on host fitness. A) Effect of symbionts on insect development rate to each life stage following inoculation. We statistically examined the effect of symbiont on rates of development to adulthood (reproductive maturity) and found insects inoculated with conspecific symbionts exhibited the most rapid development. Significant differences across treatments are indicated by letters where treatments sharing the same letter do not differ from one another. B) Effect of symbionts on the proportion of insects surviving to adulthood. Insects inoculated with conspecific symbionts exhibited higher survival to adulthood than those inoculated with the soil isolate and those lacking a symbiont (aposymbiotic) but not those inoculated with the heterospecific symbiont. Error bars show standard error around the mean across replicates and asterisks indicate significance. C) Survival probability across time. The survival probability of insects inoculated with both the conspecific and heterospecific symbionts was higher than those inoculated with the soil isolate, and there was no significant difference between the conspecific and heterospecific symbionts on insect survival probability. The survival curve for insects inoculated with the conspecific symbiont ends at 56 days, which is the time point by which all insects reached adulthood. Data were censored when insects reached adulthood, indicated by the hash marks occurring along the survival curves. Across all plots, color indicates symbiont origin: Dark Green = conspecific host symbiont, light green = heterospecific host, orange = soil, gray = aposymbiotic. ** = *p* < 0.01, *** = *p* < 0.001.

We inoculated *A. tristis* insects with Burkholderiaceae strains isolated from a conspecific host, a heterospecific host, and the soil. We then measured host development and survival until insects reached adulthood. Overall, symbiont evolutionary history had a large effect on host fitness (Fig. 1).

Symbiont origin underpinned *A. tristis* development rate to adulthood (χ^2^ = 28.44, df = 3, *P *< 0.0001). This effect resulted from insects inoculated with the conspecific symbiont strain exhibiting more rapid development to adulthood than those inoculated with the heterospecific symbiont (HR = 3.44, 95% CI [1.86, 6.36], *P *= 0.0005), the soil strain (HR = 4.43, 95% CI [2.03, 9.67], *P *= 0.0011), and aposymbiotic insects (HR = 5.73, 95% CI [2.74, 12.00], *P *< 0.0001) (Fig. 1A). In contrast, the development rates of insects inoculated with the heterospecific and soil strains did not differ from those of aposymbiotic insects, suggesting these strains offered little symbiotic benefit for development.

We then examined the effect of symbiont evolutionary history on insect survival. Symbiont origin also impacted the proportion of insects surviving to adulthood (χ^2^ = 18.68, df = 3, *P *= 0.0003; Fig. 1B), with the conspecific symbiont increasing *A. tristis* survival by 60% relative to insects inoculated with the soil isolate (*P *= 0.0025) and aposymbiotic insects (*P *= 0.0031). Although the survival of insects inoculated with the heterospecific symbiont was 37% lower than those inoculated with the conspecific symbiont, we did not observe a difference in survival to adulthood across these treatments (*P *= 0.20). However, the survival of insects inoculated with the heterospecific symbiont also did not differ from those inoculated with the soil symbiont nor aposymbiotic insects.

To gain greater insight into the effect of symbiont history on insect survival, we then directly compared survival probability over time across the 3 symbiont treatments, excluding the aposymbiotic treatment. Symbiont origin impacted insect survival probability (χ^2^ = 9.62, df = 2, *P *= 0.0081; Fig. 1C), and insects inoculated with both the conspecific (HR = 0.50, 95% CI [0.30, 0.86], *P *= 0.03) and heterospecific symbionts (HR = 0.54, 95% CI [0.34, 0.86], *P *= 0.02) exhibited higher survival probabilities than those inoculated with the soil isolate. Survival probability over time did not differ between the conspecific and heterospecific treatments (HR = 0.94, 95% CI [0.54, 1.64], *P *= 0.97). Together, our survival analyses suggest that symbionts isolated from both conspecific and heterospecific hosts both offer better support for *A. tristis* survival than the soil isolate.

Finally, we examined whether symbionts exhibited differences in within-host growth by extracting the symbiotic organs of insects that reached adulthood and quantifying the number of bacterial CFUs within each organ (see Methods). The number of CFUs per symbiotic organ was consistent across all 3 symbiont treatments (*F_2,32_* = 0.26, *P *= 0.771), with an average of ∼10^5^ CFUs/organ. This result suggests that differences in survival across treatments do not stem from differences in within-host replication rates.

## Discussion

We tested whether the evolutionary history of a symbiont impacts its quality for hosts. To do this, we inoculated *A. tristis* squash bugs with Burkholderiaceae strains isolated from a conspecific host, a heterospecific host, and soil. We found that the soil isolate offered fewer benefits to insect development and survival than both strains with a recent history of host association. In contrast, symbionts isolated from conspecific and heterospecific hosts both supported *A. tristis* survival to adulthood. Slightly increased fitness benefits emerged when insects were inoculated with the conspecific symbiont, which supported the most rapid development to adulthood.

Our results suggest that symbiont evolutionary history can influence its quality for an insect host. The heterospecific *Paraburkholderia* strain conferred greater host survival than the soil-derived *Caballeronia* strain, despite the latter being more closely related to the conspecific symbiont (Supplementary Fig. S1). Prior work has shown that *Paraburkholderia* strains can support insect development and survival but are consistently less beneficial than *Caballeronia* (Acevedo et al. 2021, Sullivan et al. 2025). Moreover, Stoy et al. (2023) demonstrate that *A. tristis* insects exhibit equivalent fitness benefits when inoculated with genetically diverse *Caballeronia* strains isolated from conspecifics across a wide geographic distribution and from closely related *Anasa andresii* (Hemiptera: Coreidae) and *Anasa scorbutica* (Hemiptera: Coreidae) hosts. Prior work has yet to demonstrate a *Paraburkholderia* strain that confers greater benefits to Coreidae hosts than a *Caballeronia* strain nor substantial variation in the fitness effects of *Caballeronia* isolates on host fitness.

In this study, *Paraburkholderia* isolated from a distantly related insect is more beneficial than a *Caballeronia* strain isolated from soil. Therefore, our results, where a host-associated *Paraburkholderia* strain conferred greater benefit than an environmentally acquired *Caballeronia* strain, suggest that symbiont effects on host fitness are not solely predicted by taxonomic identity and instead may also depend on evolutionary history. Specifically, prior selection from hosts for specific symbiotic traits may increase bacterial adaptation to the host environment, making them more beneficial than soil isolates that experience distinct selective pressures.

Overall, our results suggest that the evolutionary history of a symbiont impacts its quality to hosts, but we recognize that our study samples only a small fraction of Burkholderiaceae diversity associated with heteropterans and their environments. It is important to note that partner switching can occur through the environment, making it difficult to determine whether environmental isolates have ever experienced a prior history of host association. Moreover, all symbiont strains assessed in this study were isolated from adults, which may bias the analysis by excluding low-quality host-associated strains that do not support insect survival to adulthood. Future work incorporating additional strains, including those isolated from juvenile insects, will be necessary to comprehensively examine whether symbiont evolutionary history consistently influences insect fitness. Whether this variation in symbiont quality directly translates into costs for hosts also requires further testing. While low-quality strains may exist in the environment, insects may readily detect and avoid low-quality symbionts.

In general, much remains unknown about symbiont transmission among heteropteran insects. For example, adult *A. tristis* insects conduct fecal transmission (Villa et al. 2023), but it is unclear whether adults exhibit behaviors that facilitate parent-to-offspring symbiont transmission. Whether fecal transmission broadly occurs across heteropteran–*Caballeronia* associations and the frequency of partner switching across species also remain unknown. Finally, while *Caballeronia* can be found at low relative abundance in the soil, studies are needed to determine how environmental selection shapes the abundance and persistence of symbionts in the environment. Overall, future work focusing on the costs of environmental transmission would benefit by examining these associations in ecologically relevant environments to determine whether symbiont evolutionary history, environmental selection on symbionts, and insect behavior underpin transmission efficacy. In general, our results lay a foundation for future work by demonstrating that environmental transmission may expose insects to bacterial partners that vary in symbiotic quality.

## Supplementary Material

ieag080_Supplementary_Data

## Data Availability

All data have been uploaded to the Dryad Digital Repository (https://doi.org/10.5061/dryad.crjdfn3m8). Sanger sequences for bacterial strains used in this study can be found on the NCBI database (GAOX1 = MZ264271; Snap2a = KF931417; LS1 = PZ427496).

## References

[ieag080-B1] Acevedo TS , Fricker GP, Garcia JR, et al 2021. The importance of environmentally acquired bacterial symbionts for the squash bug (*Anasa tristis*), a significant agricultural pest. Front. Microbiol. 12:1–18. 10.3389/fmicb.2021.719112 PMC8521078 34671328

[ieag080-B2] Bennett GM , Moran NA. 2015. Heritable symbiosis: the advantages and perils of an evolutionary rabbit hole. Proc. Natl. Acad. Sci. USA. 112:10169–10176. 10.1073/pnas.1421388112 25713367 PMC4547261

[ieag080-B3] Berasategui A , Breitenbach N, García-Lozano M, et al 2022. The leaf beetle *Chelymorpha alternans* propagates a plant pathogen in exchange for pupal protection. Curr. Biol. 32:4114–4127.e6. 10.1016/j.cub.2022.07.065 35987210

[ieag080-B4] Braendle C , Miura T, Bickel R, et al 2003. Developmental origin and evolution of bacteriocytes in the aphid–*Buchnera* symbiosis. PLoS Biol. 1:E21. 10.1371/journal.pbio.0000021 14551917 PMC212699

[ieag080-B5] Bright M , Bulgheresi S. 2010. A complex journey: transmission of microbial symbionts. Nat. Rev. Microbiol. 8:218–230. 10.1038/nrmicro2262 20157340 PMC2967712

[ieag080-B6] Bronstein JL. 2001. The exploitation of mutualisms. Ecol. Lett. 4:277–287. 10.1046/j.1461-0248.2001.00218.x

[ieag080-B7] Brumin M , Kontsedalov S, Ghanim M. 2011. *Rickettsia* influences thermotolerance in the whitefly *Bemisia tabaci* B biotype. Insect Sci. 18:57–66. 10.1111/j.1744-7917.2010.01396.x

[ieag080-B8] Chomicki G , Kiers ET, Renner SS. 2020. The evolution of mutualistic dependence. Annu. Rev. Ecol. Evol. Syst. 51:409–432. 10.1146/annurev-ecolsys-110218-024629

[ieag080-B9] Cornwallis CK , van ‘t Padje A, Ellers J, et al 2023. Symbioses shape feeding niches and diversification across insects. Nat. Ecol. Evol. 7:1022–1044. 10.1038/s41559-023-02058-0 37202501 PMC10333129

[ieag080-B10] Currie CR , Wong B, Stuart AE, et al 2003. Ancient tripartite coevolution in the Attini ant–microbe symbiois. Science (1979) 299:396–388. 10.1126/science.1078155 12532015

[ieag080-B11] Frank SA. 1996. Host–symbiont conflict over the mixing of symbiotic lineages. Proc. R. Soc. Lond. B 263:339–344. 10.1098/rspb.1996.0052 8920255

[ieag080-B12] Garcia JR , Laughton AM, Malik Z, et al 2014. Partner associations across sympatric broad-headed bug species and their environmentally acquired bacterial symbionts. Mol. Ecol. 23:1333–1347. 10.1111/mec.12655 24384031

[ieag080-B13] García-Lozano M , Henzler C, Porras MÁG, et al 2024. Paleocene origin of a streamlined digestive symbiosis in leaf beetles. Curr. Biol. 34:1621–1634.e9. 10.1016/j.cub.2024.01.070 38377997

[ieag080-B14] Gerardo NM , Hoang KL, Stoy KS. 2020. Evolution of animal immunity in the light of beneficial symbioses. Phil. Trans. R Soc. B 375:20190601–20190613. 10.1098/rstb.2019.0601 32772666 PMC7435162

[ieag080-B15] Gordon ERL , McFrederick Q, Weirauch C. 2016. Phylogenetic evidence for ancient and persistent environmental symbiont reacquisition in Largidae (Hemiptera: Heteroptera). Appl. Environ. Microbiol. 82:7123–7133. 10.1128/AEM.02114-16 27694238 PMC5118923

[ieag080-B17] Herre EA , Knowlton N, Mueller UG, et al 1999. The evolution of mutualisms: exploring the paths between conflict and cooperation. Trends Ecol. Evol. 14:49–53. 10.1016/S0169-5347(98)01529-8 10234251

[ieag080-B18] Hosokawa T , Kikuchi Y, Shimada M, et al 2007. Obligate symbiont involved in pest status of host insect. Proc. R Soc. B 274:1979–1984. 10.1098/rspb.2007.0620 PMC2275188 17567556

[ieag080-B19] Junker AD , Chen JZ, Dubose JG, et al 2025. Dynamic reciprocal morphological changes in insect hosts and bacterial symbionts. J. Exp. Biol. 228:1–9. 10.1242/jeb.249474 PMC11993259 39886814

[ieag080-B20] Kaltenpoth M , Flórez LV. 2020. Versatile and dynamic symbioses between insects and *Burkholderia* bacteria. Annu. Rev. Entomol. 65:145–170. 10.1146/annurev-ento-011019-025025 31594411

[ieag080-B21] Kaltenpoth M , Goettler W, Koehler S, et al 2010. Life cycle and population dynamics of a protective insect symbiont reveal severe bottlenecks during vertical transmission. Evol. Ecol. 24:463–477. 10.1007/s10682-009-9319-z

[ieag080-B22] Kaltz O , Koella JC. 2003. Host growth conditions regulate the plasticity of horizontal and vertical transmission in *Holospora undulata*, a bacterial parasite of the protozoan Paramecium caudatum. Evolution (N. Y) 57:1535–1542. 10.1111/j.0014-3820.2003.tb00361.x 12940358

[ieag080-B23] Kikuchi Y , Ohbayashi T, Jang S, et al 2020. *Burkholderia insecticola* triggers midgut closure in the bean bug *Riptortus pedestris* to prevent secondary bacterial infections of midgut crypts. ISME J. 14:1627–1638. 10.1038/s41396-020-0633-3 32203122 PMC7305115

[ieag080-B24] Lenth RV. 2022. emmeans: Estimated Marginal Means, aka Least-Squares Means. CRAN.https://CRAN.R-project.org/package=emmeans.

[ieag080-B25] Martinez K , Stillson PT, Ravenscraft A. 2024. Inferior *Caballeronia* symbiont lacks conserved symbiosis genes. Microb. Genom 10:1–11. 10.1099/mgen.0.001333 PMC11893276 39680049

[ieag080-B26] Mendiola SY , Chen JZ, Lukubye B, et al 2024. Differential gene expression in the insect vector *Anasa tristis* in response to symbiont colonization but not infection with a vectored phytopathogen. Front. Ecol. Evol. 12:1–14. 10.3389/fevo.2024.1390625

[ieag080-B27] Oliver KM , Russell JA, Moran NA, et al 2003. Facultative bacterial symbionts in aphids confer resistance to parasitic wasps. Proc. Natl. Acad. Sci. USA. 100:1803–1807. 10.1073/pnas.0335320100 12563031 PMC149914

[ieag080-B28] Packauskas R. 2010. Catalog of the Coreidae, or leaf-footed bugs, of the New World, Fort Hays Studies, fourth aeries, Number 5. Ann. Entomol. Soc. Am. 102:278. 10.1603/RAN12001

[ieag080-B29] Parajuli BS , Teodosio J, Ravenscraft A. 2026. Leaffooted bugs enrich local soil with their horizontally acquired symbiont. Front. Microbiol. 17:1737071. 10.3389/fmicb.2026.1737071 42338892 PMC13284837

[ieag080-B31] Salem H , Florez L, Gerardo N, et al 2015. An out-of-body experience: the extracellular dimension for the transmission of mutualistic bacteria in insects. Proc. R Soc. B 282:20142957. 10.1098/rspb.2014.2957 PMC4375872 25740892

[ieag080-B32] Stoy KS , Chavez J, De Las Casas V, et al 2023. Evaluating coevolution in a horizontally transmitted mutualism. Evolution (N. Y.) 77:166–185. 10.1093/evolut/qpac009 36622711

[ieag080-B33] Sullivan LT , Kelly SE, Ravenscraft A, et al 2025. Acquisition of an obligate environmental symbiont may be limited in the arboreal environment. FEMS Microbiol. Ecol 101:1–9 10.1093/femsec/fiaf045 PMC12063585 40280734

[ieag080-B34] Therneau TM. 2024. coxme: Mixed Effects Cox Models. 10.32614/CRAN.package.coxme

[ieag080-B35] Villa SM , Chen JZ, Kwong Z, et al 2023. Specialized acquisition behaviors maintain reliable environmental transmission in an insect-microbial mutualism. Curr. Biol. 33:2830–2838.e4. 10.1016/j.cub.2023.05.062 37385254

