# Supplementary material for: Symbiont evolutionary history underpins quality to an insect host
Source: J Insect Sci. 2026 Jul 29;26(4):ieag080. doi: 10.1093/jisesa/ieag080 (PMC13420500; doi:10.1093/jisesa/ieag080)
Supplement: ieag080_Supplementary_Data [file ieag080_supplementary_data.zip › Figure S1.docx]

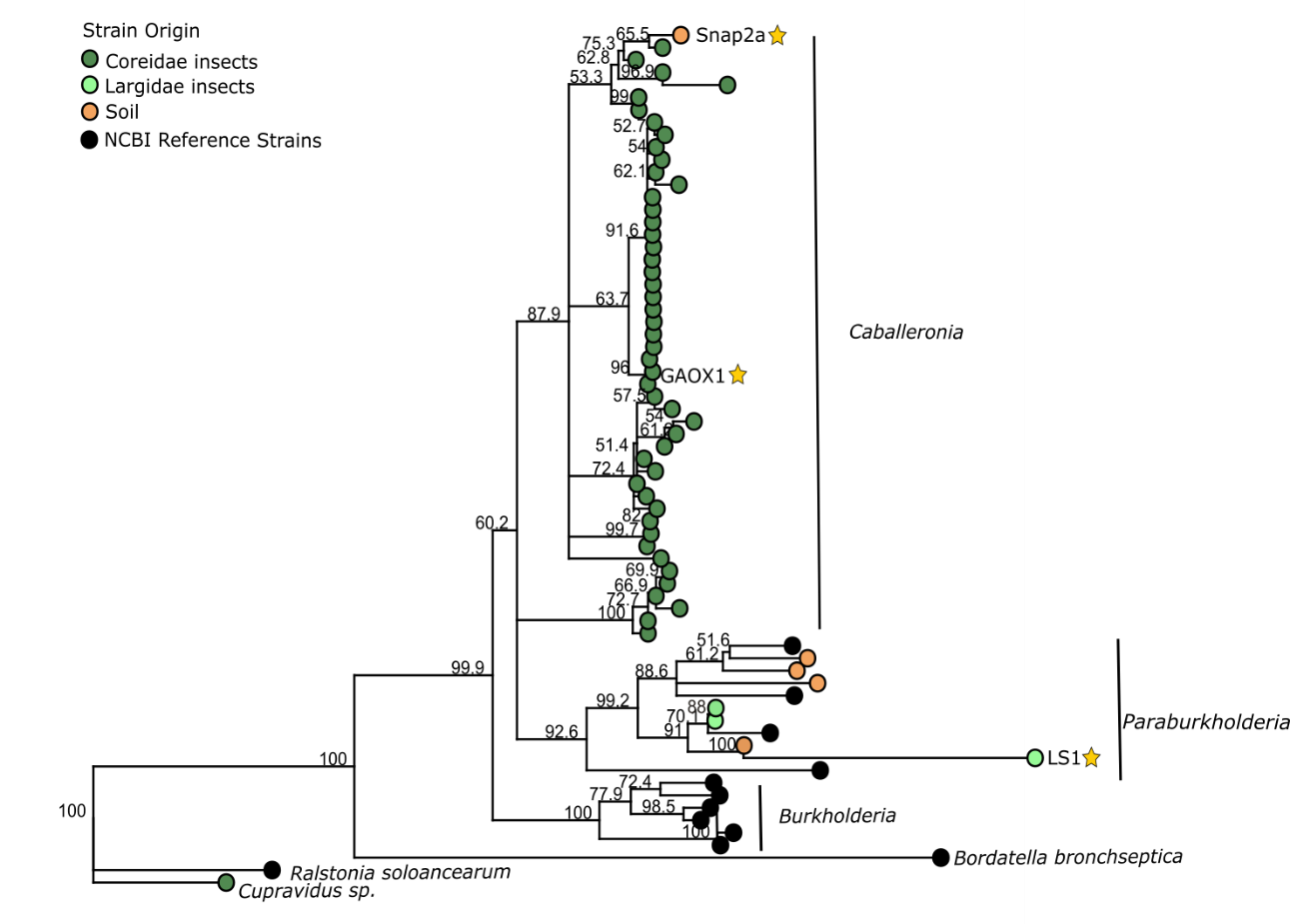


**Figure S1.** Phylogenetic confirmation of strain identities. To confirm strain identities, 16S rRNA gene sequences from isolates used in this study were compared with reference sequences from *Caballeronia*, *Paraburkholderia*, and *Burkholderia*, including bacterial isolates in our laboratory collection and publicly available sequences downloaded from NCBI. Sequences were aligned in Geneious Prime version 2025.2.2 using MUSCLE, and a neighbor-joining phylogeny was constructed with 1,000 bootstrap replicates. Colored circles at branch tips indicate strain source: dark green = Coreidae-associated strains, light green = Largidae-associated strains, orange = soil-associated strains, and black = clinical reference strains downloaded from NCBI. Bootstrap support values >50% are shown at the nodes. Strains used in this paper (Snap2a, GAOX1, and LS1) are indicated by a yellow star. All strains and accession numbers used to prepare this phylogeny are listed in Table S1.
